# Decoding the role of immune T cells: A new territory for improvement of metabolic‐associated fatty liver disease

**DOI:** 10.1002/imt2.76

**Published:** 2023-01-18

**Authors:** Jia Liu, Mingning Ding, Jinzhao Bai, Ranyi Luo, Runping Liu, Jiaorong Qu, Xiaojiaoyang Li

**Affiliations:** ^1^ School of Life Sciences Beijing University of Chinese Medicine Beijing China; ^2^ School of Chinese Materia Medica Beijing University of Chinese Medicine Beijing China

**Keywords:** drug therapy, immunoregulation, intestinal flora, metabolic‐associated fatty liver disease, T cells

## Abstract

Metabolic‐associated fatty liver disease (MAFLD) is a new emerging concept and is associated with metabolic dysfunction, generally replacing the name of nonalcoholic fatty liver disease (NAFLD) due to heterogeneous liver condition and inaccuracies in definition. The prevalence of MAFLD is rising by year due to dietary changes, metabolic disorders, and no approved therapy, affecting a quarter of the global population and representing a major economic problem that burdens healthcare systems. Currently, in addition to the common causative factors like insulin resistance, oxidative stress, and lipotoxicity, the role of immune cells, especially T cells, played in MAFLD is increasingly being emphasized by global scholars. Based on the diverse classification and pathophysiological effects of immune T cells, we comprehensively analyzed their bidirectional regulatory effects on the hepatic inflammatory microenvironment and MAFLD progression. This interaction between MAFLD and T cells was also associated with hepatic‐intestinal immune crosstalk and gut microbiota homeostasis. Moreover, we pointed out several T‐cell‐based therapeutic approaches including but not limited to adoptive transfer of T cells, fecal microbiota transplantation, and drug therapy, especially for natural products and Chinese herbal prescriptions. Overall, this study contributes to a better understanding of the important role of T cells played in MAFLD progression and corresponding therapeutic options and provides a potential reference for further drug development.

## Highlights

- Metabolic‐associated fatty liver disease (MAFLD) is the most common fatty liver disease caused by metabolic dysregulation.

- The maladjustment of T cell homeostasis including CD4/CD8^+^ T cells, Treg cells, γδ T cells, NKT cells, and MAIT cells give rise to severe hepatic steatosis and fat accumulation.

- Intestinal flora regulation and pharmacotherapy, such as traditional Chinese medicine, can restore the immune microenvironment of T cells in livers to improve MAFLD.

## Graphical abstract

Immune T cells are involved in metabolic‐associated fatty liver disease (MAFLD) progression either in a positive or negative manner and the therapeutic methods of MAFLD caused by disordered T cells include gut microbiota regulation, adoptive cell transfer, gene editor microRNAs, drugs (inhibitors/traditional Chinese medicine) therapy.

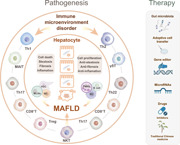

## INTRODUCTION

Metabolic‐associated fatty liver disease (MAFLD) is a progressive condition of genetic, anthropometric and metabolic dysfunction‐induced fatty liver disease, and can develop into cirrhosis and even hepatocellular carcinoma (HCC) if left untreated continuously [1]. In fact, MAFLD is a new definition that highlights the role of metabolic factors in the disease etiology instead of nonalcoholic fatty liver disease (NAFLD) since the latter does not accurately reflect comprehensive mechanisms and the severity of fatty liver diseases, and hinders the progress of medical development, to a certain extent [2, 3]. Based on plentiful clinical reports, MAFLD is now confirmed by histology, radiological imaging, or serologic detection that reflects the excessive fat accumulation in livers with metabolic dysfunction, which also increases the occurrence probability of steatohepatitis and liver fibrosis. Unlike the biopsy difficulty in the clinic, diagnostic criteria for MAFLD in animal models are usually pathological examination and the detection of blood biomarkers of fatty liver, including alanine aminotransferase (ALT), aspartate aminotransferase (AST), total cholesterol (TC) and triglyceride (TG). More shockingly, the number of MAFLD is continuously increasing and may expand to 100.9 million cases in the United States by 2030 [4], which affects up to 25% of the global population and plays a growing impact on economic development, public health, and social determinants.

There is accumulating evidence that age, unhealthy diet, metabolic status, intracellular oxidative stress, mitochondrial dysfunction, inflammatory cell infiltration, and composition of gut microbiota are the main risk factors and influence the physiopathological processes of MAFLD [1]. Nevertheless, MAFLD still encounters current dilemma due to unclear pathogenesis and the lack of FDA‐approved drugs in clinical treatment. Although there is a great deal of drugs in the pipeline that are reckoned as good candidates to cure MAFLD [5], most of them mainly focus on the activation of PPARγ or GLP‐1 receptor and insulin sensitization with several serious side effects [6]. With the deepening of research, another condition that plays a crucial role and is greatly associated with the pathogenesis of MAFLD is the dysfunction of immunomodulatory T cells. For instance, Van Herck et al. found that CD4^+^ T cells and CD8^+^ T cells sharply increased and abnormally secreted proinflammatory factors such as interferon‐γ (IFN‐γ) and tumor necrosis factor α (TNF‐α) with the occurrence of MAFLD [7]. According to another report, immune homeostasis in the mouse model of MAFLD is broken, as shown by unbalanced CD4^+^ T cells subsets and excessive secretion of pathogenic cytokines, specifically IFN‐γ and interleukin‐17A (IL‐17A) [8]. Accordingly, understanding the intricacies of T cells involved in MAFLD progression may provide new avenues for therapeutic intervention.

## T CELL‐SPECIFIC IMMUNE RESPONSES

**Figure 1:**
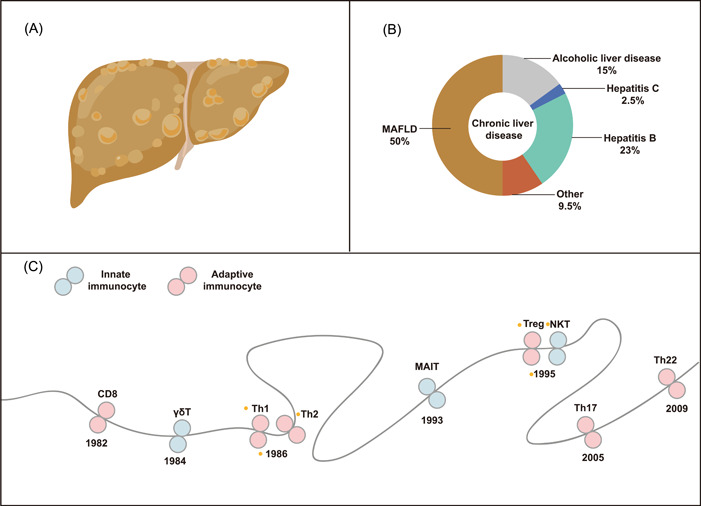
The general background summary of MAFLD and immunocytes. (A) The liver of MAFLD. (B) The proportion of prevalence of common liver diseases. (C) Timeline of T cell development. MAFLD, metabolic‐associated fatty liver disease.

The immune system is a collection of cells and molecules that mediate immune responses, such as allergy, tumor immunity, and autoimmunity, which can be roughly divided into innate immunity and adaptive immunity. Among them, T cells are differentiated from pluripotent stem cells (PSCs) within the bone marrow and spread throughout the body for coordinating the homeostasis of the immune system. T‐cell receptors (TCRs) are composed of the α and β protein (αβ T cells) with a T cell‐specific coreceptor CD3 complex, which recognizes the specific signals to initiate cellular biological responses [9]. Naive T cells have been established as a crucial component of the thymus‐related immune response, once under antigen stimulation, will exit the quiescence state into the clonal expansion and differentiation state. Furthermore, the differentiation of T cells recognizes and binds to the major histocompatibility complex (MHC) molecules of antigen‐presenting cells (APC) by TCRs to ensure that the immune responses generate though the specifically recognizing antigens [10]. Besides, T cells can be subcategorized into CD4^+^T helper cells, CD8^+^ cytotoxic T cells and innate T cells, including γδ T cells, natural killer T (NKT) cells, and mucosa‐associated invariant T (MAIT) cells [11] (Figure 1).

### Physiological characteristics and functions of different T cells

CD4^+^ T cells, namely T helper (Th) cells, recognize peptides binding to Class II MHC molecules to differentiate into subsets of effector cells, stimulating different cytokines and performing distinct functions [12]. Under the stimulation of microorganism antigens and/or the cytokines IL‐12 and IFN‐γ, CD4^+^ T cells are stimulated and start to differentiate into Th1 cells, which are also able to secrete cytokines including IL‐2, TNF‐α, and IFN‐γ as the first defined subset of helper T cells [13]. As the signature cytokine of Th1 cells, IFN‐γ not only affects the development and function of Th1 cells but also leads to the subsets of Th1 cell polarization when organisms are invaded by pathogens including fungus, bacteria (especially intracellular bacteria) and viruses [14]. Th1 cells play an antifibrotic role by secreting a large amount of IFN‐γ to reduce collagen synthesis in fibroblasts [15]. On the other hand, Th1 cells and IFN‐γ may also induce transforming growth factor‐β (TGF‐β) expression in myofibroblasts, suggesting that the immunomodulatory effects of Th1 cells are complex and need further investigation [16]. The differentiation of Th2 cells is controlled by transcription factor GATA3 or IL‐4, which mainly releases a variety of different cytokines like IL‐4, IL‐5, and IL‐13 through the regulation of activator of transcription 5 (STAT5) and STAT6 [17]. It is worth to note that Th2 cells‐released cytokines such as IL‐4 and IL‐13 can interfere Th1 cell‐mediated immune reactions and alternate classical macrophage activation against intracellular infection [14]. Th2 cells induced hepatic fibrosis by inhibiting Th1‐associated IFN‐γ expression levels. In addition, IL‐13 secreted by Th2 cells could also cause liver fibrosis by activating hepatic stellate cells (HSC) [18]. These evidence point out that Th1 cells and Th2 cells are associated with multiple disease processes and their balance directly affects the progression of various diseases. Th22 cells, also belonging to the subset of helper T cells and adjusted by IL‐6, TNF‐α, specifically secrete IL‐22 and inhibit the presence of TGF‐β and its differentiation. In the liver, Th22 cells are involved in the processes of wound healing, tissue repair and cell regeneration by expressing the fibroblast growth factor (FGF) [19]. Interestingly, plenty of observations indicated that IL‐22 could be released by Th22 cells and even other immune cells, including Th1/Th17 cells, CD8^+^ T cells, NKT cells as well as some nonlymphocytes [20]. In addition to cellular interregulation of Th1 and Th2 cells mentioned above, Th17 cells and regulatory T (Treg) cells are another important pair of immune cells that are derived from T lymphocytes and naive T cells, respectively. Served as an extremely proinflammatory cells, Th17 cells can be irritated and then start their differentiation under the activation of crucial transcription factors retinoic acid receptor‐related orphan receptor γt (RORγt) and STAT3, and further promote the secretion of IL‐17, IL‐22, IL‐23 and inflammatory response [21]. The abnormal increase of Th17 cell frequency may be related to TGF‐β1/IL‐17 in chronic hepatitis B (CHB) and hepatitis B virus (HBV)‐associated liver cirrhosis patients [22]. However, unlike other Th cells with CD4^+^ positive phenotype, Treg cells mainly exert the immune‐controlling effects to maintain the balance of the immune system by the activation of forkhead box P3 (Foxp3), producing the inhibitory cytokines of IL‐10 and TGF‐β [23]. Additionally, Treg cells restrain immune responses and regulate self‐tolerance by several mechanisms; but once lack of FOXP3 gene, dysfunction of natural Treg cells causes host self‐intolerance and lead to a variety of immune dysregulation‐linked syndromes [24]. Additionally, research reported that Treg depletion caused high activation and concentration of CCR2^high^ Ly‐6C^high^ monocytes/macrophages and the profibrotic Th2 cells to exacerbate liver inflammation [25]. Compared to the control group, the higher quantity of Tregs can combat the hepatic injury and inflammation in patients with self‐tolerant, autoimmune, and virus‐related liver disease [26]. Treg cells are also involved in the progression of various inflammatory diseases including type 1 diabetes, MAFLD and allergic diseases but the pathogenesis of these diseases remain to be further explored. Beyond CD4^+^ T cells, the subset of CD8^+^ T cells proliferate and differentiate into cytotoxic T lymphocytes (CTLs) and memory cells by the costimulation of MHC‐I and CD4^+^ T cells or CD8^+^ T cells‐derived IL‐2 [27]. CD8^+^ CTLs secrete IFN‐γ and activate the classical macrophage immune response to kill extracellular microorganism [14]. On the one hand, CD8^+^ CTLs have the functional capacity to initiate the apoptosis of infected cells by releasing granzymes and perforin [27]; on the other hand, they also cooperate with CD4^+^ T cells to eradicate intracellular infections and cancer cells by recognizing Class I MHC‐associated peptides on tumor cells [12]. Lan Baudi et al. reported that specific CD8^+^ T cells responses had a central role in virus clearance and were restrained by chronic antigenic stimulation or the loss of costimulatory or cytokine signaling in the mouse model of chronic hepatitis B [28].

Additionally, these are several small populations of T cells distinguished from CD4^+^ T cells and CD8^+^ T cells since they recognize nonprotein antigens without the involvement of Class I or Class II MHC molecules. These populations (γδ T Cells, NKT cells, and MAIT cells) all respond to infections in ways as well as are characteristic of adaptive immunity but also have features of innate immunity such as rapid responses and limited diversity of antigen recognition. The γδT cells possess innate immune function of T cells including secreting cytokines and killing infected cells [29]. The TCRs on γδT cells are able to recognize various types of protein and nonprotein antigens and help γδT cells initiate immune responses to recognize microbes before the activation of antigen‐specific T cells [30]. Intriguingly, although previous studies majorly focused on the function of γδT cells in the skin and intestine, γδT cells but not Th17 cells also can produce IL‐17 in liver and participate in liver disease progression [31]. Second, NKT cells express αβTCRs with limited diversity and balance the connection between innate and adaptive immune responses. They can not only interact with other immune cells by producing many inflammatory cytokines like IFN‐γ, IL‐4, IL‐13, and IL‐17 after antigen recognition, but also directly kill infected cells by rapidly secreting effector molecules including perforin and Fas‐ligand [32], ultimately, participating in and influencing the process of cardiovascular diseases, autoimmune disease, and cirrhosis. NKT cells can also induce the aggregation of inflammatory cells by rapidly secreting proinflammatory cytokines and chemokines to aggravate liver injury in alcoholic liver disease, MAFLD, and hepatocellular carcinoma [33]. Last but not least, MAIT cells are charactered by CD4^−^CD8^−^ (double negative) T cells or CD161^+^ and IL‐18Rα^+^ CD8αβ T cells, mainly homing to intestine and liver, and are genetically conserved T cell subset [34]. Most MAIT cells can express high levels of multidrug resistance transporter (ABCB1) and αβ TCRs with limited diversity, and produce IFN‐γ, granzyme‐B, and IL‐17 [35]. Furthermore, MAIT cells are rapidly activated and highly enriched to modulate the host defense and inflammation in the liver by the abundant expression and release of IFN‐γ and TNF‐α [36]. It is worth noting that MAIT cells promote tissue repair in the liver, and its absence will initiate or exacerbate injuries, suggesting that MAIT cells are emerging therapeutic targets for improving liver disorders [37].

Collectively, growing evidence suggest that Th cells subset, Treg cells, CD8^+^ T cells and innate T cells are involved in the pathogenesis of inflammation and steatosis. Meanwhile, T cells are attractive therapeutic targets for immune regulation in a variety of diseases including MAFLD, autoimmune hepatitis (AIH) [26], hepatic ischemia‐reperfusion injury (HIRI), and liver fibrosis [38]. What is noticeable is that it is very urgent to clarify the heterogeneity and pathogenesis and look for new therapeutic target of MAFLD, nevertheless the relationship between immunity and MAFLD has been overlooked. Considering the fact of bottleneck problem such as medicine deficiency and the importance of immunoregulation, we comprehensively summarize the literature about the relationship and contradictory roles of T cells played in MAFLD and try to provide a new therapeutic direction for the treatment of MAFLD and related complications.

## T CELLS AND MAFLD

### Effects of Th1 cells on MAFLD

As classical proinflammatory cells, Th1 cells either rigger the phagocyte‐mediated host defense by inducing costimulatory molecule CD40L‐CD40 interactions or releasing IFN‐γ, or regulate cellular immunity‐specific homeostasis to ingest or against surviving pathogens in macrophages. Given these effects, Th1 cells are closely relevant to macrophages‐mediated inflammatory reaction in the pathogenesis of MAFLD. In livers of obese patients with MAFLD, elevated hepatic immune semaphorins stimulated the activation and differentiation of Th1 cells and further induced Th1 cells and macrophages to secrete proinflammatory cytokines to cause a low‐grade inflammation without histological abnormalities [39]. Sutti et al., further investigate the action and potential mechanism of Th1 cells played in the MAFLD progression. They first demonstrated that methionine‐choline deficiency (MCD) diet promoted lobular inflammation and MAFLD injury accompanied with the increase of immunoglobulin G (IgG) against malonyldialdehyde (MDA)‐derived antigens. Once immunized with MDA‐adducted bovine serum albumin (BSA) before MCD feeding, mice suffered more severe steatohepatitis due to the recruitment and activation of Th1 cells, and subsequently, M1 macrophages [40]. Furthermore, the high‐fat diet (HFD) also resulted in an elevation of T cells numbers, especially for Th1 cells [41]. It has been well‐recognized that monocyte chemoattractant protein‐1 (MCP‐1) triggered or heightened the expression of inflammatory factors/cells whereafter participated in the process of steatosis [42]. Notably, IFN‐γ deficiency decreased the HFD‐induced accumulation of inflammatory cells and cholesterol leptin levels and improved insulin sensitivity by reducing the expression of inflammatory factors TNF‐α and MCP‐1 in the adipose tissues of mice [41]. Another study found that MAFLD and multiple inflammatory response could be improved by inhibiting Th1 cells‐ or their pivotal cytokines‐related receptors like CXCR3 [43]. Zhang et al. further proved the importance of this receptor and discovered that the deficiency of CXCR3 ameliorated fat accumulation, steatohepatitis and MAFLD by attenuating Th1 cells‐induced immune response and reducing the secretion of the cytokine IFN‐γ and inhibiting NF‐κB activation in the MCD‐induced mouse liver [44].

In addition to the increased Th1 cells alone, increasing evidence also found that Th1 cells and a variety of other immune cells could cooperate together to regulate inflammatory response and MAFLD progression. The tumor necrosis factor receptor OX40 (CD134) is expressed on the activated T cells as a T cell costimulatory molecule [45]. With HFD‐fed irritation, knockout of OX40 remarkably inhibited fat accumulation, lobular inflammation and focal necrosis by inhibiting Th1 cells' differentiation and proliferation, restraining monocyte migration and M1 macrophage polarization and restricting the expression of proinflammatory cytokines, such as IFN‐γ and TNF‐α [46]. More interestingly, B2 lymphocytes accelerated steatohepatitis progression through the interaction with the activation of Th1 cells in the early stage of MAFLD [47]. After lipopolysaccharide (LPS) and anti‐CD40/IgM stimulation, intrahepatic B (IHB) cells aggravated hepatitis and MAFLD by inducing the secretion of IL‐6, TNF‐α, and IgG2a and enhancing the differentiation and aggregation of Th1 cells in the HFD‐induced MAFLD model [48]. Thus, the interplay between either T cells and B cells or other nonparenchymal cells complexly influences the progression and development of MAFLD.

In addition, although MAFLD and HCC are completely different phenotypic diseases, the metabolic‐associated steatohepatitis has been become the rapidly increasing cause of hepatic malignant tumors [49]. The opinion is supported by clinical observations, showing that western diet‐induced mice sequentially developed MAFLD, progressive fibrosis, and spontaneous HCC as time goes by. During this process, Th1 inflammatory pattern dominated and maintained the systemic inflammation while facilitated HCC development, rather than relying on the Th17 cells inflammatory pattern [49]. Hence, Th1 cells play a significant role in the entire process of MAFLD as well as the later malignant liver disease.

### Effects of Th2 cells on MAFLD

Th2 cells effectively improve inflammatory levels of visceral and subcutaneous adipose tissue by secreting IL‐4, IL‐5, and IL‐13 [50]. After being fed with HFD, obese Alms1 mutant (foz/foz) C57BL6/J mice developed severe MAFLD and liver fibrosis, accompanied by increased ratio of IL‐4 and IFN‐γ, higher Th‐2 predominance and upregulated expression of platelet‐derived growth factor (PDGF)‐α and connective tissue growth factor (CTGF) in livers [51]. Later on, researchers continued to investigate this phenomenon and attributed it to the highly polarized Th2 immune response. Accumulated Th2 cells could secrete a large amount of IL‐4 and IL‐13 to facilitate the release of growth factors from macrophages such as PDGF‐α and CTGF, and directly activate fibroblasts, eventually launching fibrosis in injured liver tissues [18, 50, 52]. Besides to classic Th2‐related cytokines, it is noteworthy to note that IL‐33 was emerging as another crucial immune modulator that released signals to alert immune cells upon cell injury or tissue damage in chronic inflammatory diseases [53]. Data from MAFLD patients and HFD‐ or MCD‐fed mice showed that, on the one hand, IL‐33 was outstandingly high in the serum and liver of patients with MAFLD and significantly activated Th2 cells and hepatic M2 macrophages, recruited Th2‐cytokines like IL‐4, IL‐5, and IL‐13 and downregulated IFN‐γ level to reverse macrovesicular steatosis, and eventually, improved MAFLD symptoms in the dose‐dependent manner [54]. However, on the other hand, the highly expressed IL‐33 resulted in chronic tissue injury and even enhanced liver fibrosis in the HFD‐fed mice via responding to the fibrosis signaling of tumorigenicity 2 receptor (ST2)/IL‐13, which could be improved by knocking out galectin‐3 (Gal‐3) [55]. Collectively, the connections between the immune microenvironment and MAFLD need an integrated view.

### Effects of Th1 cells‐Th2 cells interaction on MAFLD

**Figure 2:**
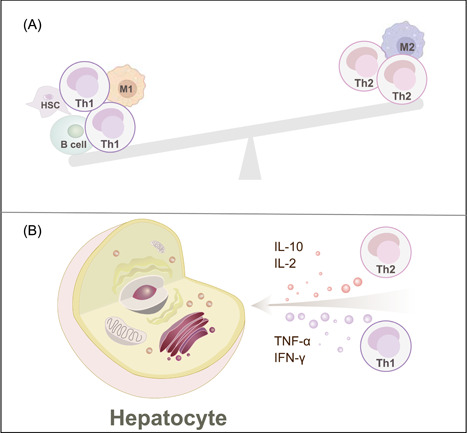
The homeostasis of Th1‐Th2 cells plays a decisive role in the progression of MAFLD. (A) Imbalance of Th1 and Th2 cells is accompanied by multicellular crosstalk. (B) Hepatocytes are impacted by disordering the proportion of Th1 and Th2 cells and their crucial inflammatory factors in MAFLD. Th1 cells upregulated the level of inflammatory cytokines and receptors, exacerbated hepatitis and lipid deposition, and even participated in the process of HCC. In addition, Th2 cells antagonized Th1 cells response and inhibited inflammation through anti‐inflammatory cytokines.

Considering the antagonistic effects of Th1 cells and Th2 cells on the regulation of immune response, more and more researchers attempt to investigate the pathogenesis of MAFLD by regulating Th1 cells‐Th2 cells balance as the break though point (Figure 2). Clinical data demonstrated severe symptoms of inflammation in the peripheral blood and hepatic vein (HV) of obese MAFLD patients due to the maladjustment of Th1 cells‐Th2 cells homeostasis. In the liver of mild, moderate, and severe MAFLD patients, the systemic proinflammatory Th1 state was enhanced while the anti‐inflammatory Th2 pathway was also stimulated to counterbalance the upregulation of Th1 cells [56]. HFD significantly raised the levels of TG, TC, low‐density lipoprotein (LDL), malonaldehyde (MDA) and the numbers of Th1 cells and declined the levels of high‐density lipoprotein (HDL), nicotinamide adenine dinucleotide (NAD), and the numbers of Th2 cells in the rat liver, suggesting a mutually restrictive relationship between Th1 cells and Th2 cells. Koumine, an ingredient isolated from *Gelsemium elegans*, was found to reverse the above situation and also improve steatohepatitis by inhibiting the differentiation of the Th1 cells and the secretion of cytokines IFN‐γ and TNF‐α, and increasing the production of Th2 cells and anti‐inflammatory cytokine IL‐10 [57]. The toll‐like receptor 9 (TLR9), a family of pattern recognition molecules and typical inflammatory receptor, also contributes to inflammatory infiltration and proinflammatory process activation in MAFLD [58]. As proved by evidence, the knockout of TLR9 inhibited the recruitment of inflammatory cells including M1‐type macrophages and Th1 cells and upset the balance of Th1 cells and Th2 cells to restrain lobular inflammation and MAFLD progression through the reduction of Th1 cells‐released proinflammatory cytokines including MCP‐1 and TNF‐α and the augmentation of Th2 cells‐released anti‐inflammatory cytokines to promote predominant M2 polarization in atherogenic (Ath)‐fed mice [59]. It is well known that transcription factors such as T‐bet and GATA‐3 can regulate T cell differentiation and control cell proliferation. After concanavalin A (ConA) stimulation, the aggravation of hepatitis was mainly attributed to the intensification of Th1 response and increased Th1 cells‐released cytokines like TNF‐α, IL‐12 and IFN‐γ by upregulating the expression of Th1 cells‐related transcription factor T‐bet, while inhibiting the level of the Th2 cells‐related transcription factor GATA‐3 in choline‐deficient diet (CD)‐induced mice [60]. α‐GalCer, as an activator of NKT cells, can be specifically recognized by invariant natural killer T cells (iNKT) [61]. Intraperitoneal injection of α‐GalCer strongly caused the upregulation of iNKT2 quantity by increasing GATA‐3 while decreasing the expression of T‐bet to reverse hepatitis in HFD‐induced MAFLD [61].

Restoring immune homeostasis through drug therapy is becoming a research hotspot. Jiang et al. expounded that the flavonoids of the peel of *Citrus changshan‐huyou* (Qu Zhi Ke [QZK]), total flavonoids of QZK (TFCH) not only significantly inhibited the activities of Th1/Th2 cells‐related inflammatory cytokines but also suppressed the signal transduction of NF‐κB/MAPK signaling to reduce systemic and intrahepatic inflammation and improved MAFLD in HFD‐diet mice [62]. As the research further develops, intestinal immune disorders have become one of the prime causes of MAFLD. The *probiotic Clostridium butyricum* B1 (CB), a butyrate‐producing probiotic, modulated the proliferation and differentiation of T cells, attenuated enterohepatic immunologic dissonance and steatohepatitis via promoting Th2 cells production and inhibiting Th1 cells differentiation [63]. Another piece of evidence suggests that gut microbiota is related to the differentiation and proliferation of T cells in the intestine and mesenteric lymph nodes (MLNs), a component of gut‐associated lymphoid tissue (GALT) [64]. Interestingly, except for livers, the percentage ratios of Th1/Th2 cells and Th17/Treg cells were also imbalanced in MLNs isolated from HFD‐fed mice model. These MLN CD4^+^ T lymphocytes manifested a tendency to migrate to the liver for accelerating inflammatory reactions and lipid accumulation; while the administration of antibiotics (neomycin and polymyxin B) or probiotics (*Lactobacillus*) significantly alleviated MAFLD by improving intestinal bacteria and restoring the balance of CD4^+^ T lymphocytes in MLNs [65]. Collectively, strategies adjusting Th1–Th2 cells balance to improve MAFLD might be through the preservation of gut microbiota homeostasis.

### Effects of Th22 on MAFLD

Th22 cells, originating from peripheral blood T cells and CD4^+^ T cells, can activate innate immunity of epithelia, clear away bacterial infections located on the body surfaces and specifically secrete IL‐22 [20]. MCD‐induced MAFLD mice showed the extensive infiltration of Th1 cells, Th17 cells and Th22 cells and hepatic fat accumulation by inducing c‐Jun n‐terminal kinase (JNK). Previous studies have reported that phosphoinositide 3‐kinase (PI3K)/protein kinase B (Akt) can restrain the activation of JNK but be negatively regulated by phosphatase and tensin homologue deleted on chromosome 10 (PTEN) signaling [66]. Adenosine A2a receptor (A2aR), a significant modulator of immune lipotoxicity, recognizes and binds adenosine to activate the immunosuppressive environment by modulating CD4^+^Th cells response. Meanwhile, A2aR agonists CGS21680 inhibited the recruitment and polarization of Th22 cells and Th17 cells via the reduction of CXCL10 and CCL2, restrained JNK‐dependent lipotoxicity by stimulating PI3K/Akt signaling and eventually suppressed MAFLD development in MCD‐diet mouse [66]. A subsequent study further supplemented the mechanism of how to improve lipotoxicity by upregulating IL‐22 and Th22 cells in MAFLD. IL‐22 prevented the increasing trend of JNK pathway‐related hepatocyte lipotoxicity caused by IL‐17 in hepatocytes treated with palmitate; but it is noteworthy that Th22 cells decreased JNK activation and PTEN expression, as well as increased Akt phosphorylation to alleviate inflammation and inhibit fat toxicity by transporting IL‐22 into liver only in IL‐17^−/−^ mice [67].

### Effects of Th17 on MAFLD

Not only Th22, but plenty of observations show that Th17 cells are also able to secrete IL‐22 and produce other cytokines like IL‐17 and IL‐23 to promote hepatic inflammation and enhance adaptive immune responses by recruiting inflammatory cells such as neutrophils and macrophages [68]. Notably, compared with healthy controls, infiltrated Th17 cells and upregulated Th17 cell‐related genes like ROR γt, IL‐17, IL‐21, and IL‐23 were highly increased in the livers of patients with MAFLD [69]. Notably, IL‐17 axis including IL‐17A, IL‐17F, and IL‐17A receptor (IL‐17RA) actively participated in and even exacerbated hepatic infiltration of T cells and macrophages during the progression of MAFLD and steatohepatitis [70]. Deficiency of hepatic lipase (HL) that hydrolyzed TGs and phospholipids from lipoproteins also caused hepatic steatosis and inflammation by increasing circulating inflammatory cells, including islet cells, CD4^+^ T cells and Th17 cells subsets expressing IL‐17, as well as upregulating inflammatory stress p38‐ and JNK‐signaling kinases in high‐fat/high‐cholesterol (HFHC) mice [71]. In addition, it has been pointed out that elements of oxidative stress including nicotinamide adenine dinucleotide phosphate oxidase 2 (NOX2) and reactive oxygen species (ROS) are implicated in steatosis and MAFLD pathogenesis. In addition, knockout NOX2 significantly improved the intrahepatic inflammatory environment and obviously decreased the level of CD4^+^ T cells, especially for Th17 cells and proinflammatory cytokines (IL‐17A, IFN‐γ, and TNF‐α) in HFD‐diet mice [72]. In consideration of the vital function of IL‐17, researchers have also attempted to reverse MAFLD by inhibiting IL‐17. As expected, neutralization of IL‐17 by anti‐IL‐17 antibody ameliorated the trends of liver inflammation, reduced the infiltrations of inflammatory Th17 cell and fatty degeneration via inhibiting IL‐17/insulin‐signaling pathway in HFD‐ and HFD/LPS‐induced mouse model [69]. Similarly, in the MCD‐induced MAFLD mice, cryptotanshinone (CTS), *a major* component derived from the traditional medicinal herb *Salvia miltiorrhiza* Bunge, restrained Th17 proliferation and IL‐17A production to specifically inhibit NOD‐like receptor family pyrin domain containing 3 (NLRP3) inflammasome‐mediated inflammation [73]. Based on the network pharmacology‐approach, Luo et al. predicted the active ingredients of Fuzi Lizhong Decoction (FLD), a traditional Chinese medicine (TCM) formula, and found that these ingredients could directly regulate Th17 cell differentiation, inhibit IL‐17 signaling pathways and further inhibit MAFLD progression [74]. Interestingly, adoptive cell transfer (ACT) is a biotherapy method that passively transfers immune cells into unimmunized individuals to perform therapeutic effects. The ACT of double negative CD4^+^ T cells alleviated HFD‐induced MAFLD by inhibiting the production of TNF‐α and IL‐17 and increasing the apoptosis of M1 macrophage and CD4^+^ T cells including Th17 cells [75].

Increasing studies have begun to explore whether transcription factors, critical receptors or other transcription regulatory factors are also involved in the differentiation and recruitment of Th17 cells. Serving as a secondary costimulatory immune checkpoint molecule, OX40 was found to promote the proliferation and differentiation of Th17 cells by upregulating transcription factor RORγt in the HFD‐, MCD‐, and CD‐induced MAFLD mouse models. Meanwhile, OX40^−/−^ inhibited the activation and differentiation of CD4^+^ T cells into Th17 cells and alleviated liver inflammation and MAFLD development [46]. On the other hand, emerging evidence revealed that the inflammatory element CXCR3 influenced the MAFLD progression not only by regulating of inflammatory Th1 cells‐related immune response but also by controlling Th17 cell recruitment and cytokine IL‐17 production [44]. Based on the latest discovery, the inflammatory hepatic Th17 (ihTh17) cells are a special Th17 subset that can also highly express CXCR3 in the steatotic livers. Moreno‐Fernandez et al. further reported that HFD stimulation caused accumulation of hepatic ihTh17 cells and production of various proinflammatory cytokines including IL‐17A, IFN‐γ, and TNF‐α and exacerbated liver steatosis through the activation of CXCR3/CXCL9/CXCL10 axis, which could be revised by glycolysis inhibitor, 2‐deoxy‐D‐glucose (2‐DG) or cell‐specific deletion of genes encoding pyruvate kinase M2 (PKM2) [76]. Except for the aforementioned transcription factors or receptors, microRNA can also directly regulate the transcription of related genes and then to influence IL‐17/Th17 cells‐related immune response. Previous study demonstrated that mastiha, a natural supplement with active phytochemicals, could regulate circulating microRNA‐155 that prevented liver X receptor (LXR)/lipogenic genes expression by regulating sterol regulating element binding protein 1c (SREBP‐1c) pathway and influenced Th17 cells differentiation in MAFLD patients [77].

Recent studies have found that obesity‐related intestinal dysregulation also contributes to MAFLD development by increasing intestinal microbiota products and activating proinflammatory signaling pathways. Endotoxin, the product of gut microbes, profoundly increased intestinal permeability to deteriorate the systemic immunity, and abnormally raised in the serum that was positively correlated with an increase of Th17 cells and IL‐17 in MAFLD patients [78]. Unexpectedly, IL‐17 deficiency (*IL17* ^*−/−*^) still caused MAFLD exacerbation and gut flora variation including the increase of pathogenic microorganisms (*Staphylococcaceae, Enterococcaceae*, and *Enterobacteriaceae*) and the decrease of beneficial microorganisms in MCD‐treated mice. Meanwhile, fecal microbiota transplantation (FMT) from wild‐type (WT) mice or antibiotic (Abx)‐treated mice could improve intestinal permeability and reestablish intestinal barrier to repair MCD‐caused MAFLD in *IL17* ^*−/−*^ mice. CD4^+^ T cells isolated from Il17^−/−^ mice were transplanted into Rag1^−/−^ mice, and aggravate intestinal barrier dysfunction and hepatic steatosis in recipient mice, suggesting that IL‐17 originating from CD4^+^ Th17 cells was crucial for maintaining intestinal barrier integrity in an MCD diet‐fed murine model [79]. Therefore, a connection between gut microbiota and MAFLD illustrated that gut dysbacteriosis might represent a risk factor for MAFLD. Besides, compared with the general population, HIV‐positive individuals were more susceptible to MAFLD and showed downregulation of IL‐17A and upregulation of sCD14 and IL‐22 in plasma [80]. Since sCD14 and IL‐22 may be markers of intestinal injury and endotoxin exposure, it is speculated that a key risk factor for the high incidence of MAFLD in HIV patients may be the simultaneous dysregulation of cellular immunity and intestinal flora.

At present, adipose tissue has been regarded as an important endocrine system that affects insulin sensitivity and contributes to inflammation and steatosis, instead of simply being an energy storage organ [81]. Clinical study showed that insulin‐resistant obese patients manifested as the significant proliferation of Th17 cells and Th22 cells in adipose tissues as well as high expression of IL‐17 and IL‐22 receptors in livers due to the increase of circulating IL‐22 and IL‐6 and adipose tissue‐released cytokines. In vitro, IL‐17 and IL‐22 inhibited glucose uptake in the soleus muscle of rat and decreased insulin sensitivity in human hepatocytes by restraining Akt phosphorylation, leading to metabolic dysfunction in the liver and muscle [82]. Visceral adipose tissue of high‐fat high‐fructose diet (HFHFD)‐fed mice upregulated the proportion of CD8^+^ T cells and Th17 cells but downregulated Treg cell quantity via the enrichment of the cytotoxic T‐lymphocyte‐associated protein 4 (CTLA4) signaling pathway and increase of RORγt expression. The antibody against CD8a attenuated steatosis and fibrosis by the suppression of CD8^+^ T cells proliferation in visceral adipose tissues and livers without altering the levels of Th1 cells, Th17 cells or Treg cells. On the other hand, unlike anti‐CD8a antibody therapy, anti‐IL‐17A antibody only improved intrahepatic inflammation but did not reverse other features of MAFLD [83]. A recent study showed that the treatment of PsTag600‐FGF21 (as new drug consisted of the recombinant polypeptide PsTag and the growth factor FGF21) could attenuate choline‐deficient high‐fat diet (CD‐HFD)‐induced fat accumulation, inflammatory macrophages infiltration and indirectly inhibit the proliferation of Th17 cells and IL‐17A expression by upregulating the production of adiponectin [84].

Indeed, MAFLD is not known as a simple chronic hepatic disorder but also can deteriorate even further and develop into liver fibrosis, which is a critical step of degenerating to cirrhosis and HCC. MAFLD and its related malignant liver disease are driven by special circumstances including but not limited to genetic/epigenetic changes, immune cells or signaling pathway alterations. Among these manners, epigenetics refers to the change of gene expression level through nongene sequence changes, such as DNA methylation or histone modifications, which requires the assistance of DNA methyltransferase (DNMT) and histone deacetylase (HDAC) [85]. It has been demonstrated that patients with MAFLD and cirrhosis presented with the increased number of Th17 cells, aberrant activation of HDAC2 and DNMT1, and specific expression of vascular gene IGFBP7 and ADAMTS1 (marker maladapted epithelial cells) in livers [86]. Meanwhile, the western diet and carbon tetrachloride breeding also resulted in lipid accumulation as well as the migration and recruitment of Th17 cells, which was alleviated after the combination therapy of HDAC2 inhibitor (HDAC2i) and DNMT1 inhibitor (DNMT1i). As expected, hdac2^iΔEC^ mice showed a decrease of hepatic Th17 number and ADAMTS1 expression after DNMT1i therapy; similarly, IGFBP7^−/−^ mice also showed reduction of Th17 inflammatory reaction and hepatic fibrotic response due to the absence of IGFBP7 [86]. This result demonstrated that the HDAC2/DNMT1 and IGFBP7/ADAMTS1 axis jointly affected the proliferation and differentiation of Th17 cells to deteriorate MAFLD. Hepatic unconventional prefoldin RPB5 interactor (URI) was increased under the stimulation of overnutrition caused by HFD, CD‐HFD and MCD‐HFD in livers, but heterozygous (URI(+/Δ) ^hep^) mice showed the decline of inflammatory cell infiltration and MAFLD, even with dietary interventions. The knockin of human URI (hURI‐tetOFF^hep^) mice (namely mutants), serve as spontaneous HCCs model with multistep HCC‐dependent genotoxic stress, showed the increase of neutrophils and Th17 cells in WAT by activating URI‐dependent IL‐23/IL‐17A axis [87]. Meanwhile, digoxin or IL‐17A antibody reduced the levels of neutrophils and Th17 cells, blocked IL‐17A signaling and restrained dysplastic foci (HCC precursors) by inhibiting URI to improve HCC accompanied with MAFLD [87].

### Effects of Treg cell on MAFLD

Treg cells, developed in the thymus or peripheral tissues, restrain excessive effector T cell responses and subsequent tissue inflammatory injury. The disorder of immune cells proportion, especially for Treg cells, is involved in the development of diet‐induced glucose and lipid metabolic dysregulation and MAFLD. Lian et al. indicated that oral administration of the anti‐TNF fusion protein (PRX‐106) restored the distribution of immune cells, including the decrease of hepatic CD4^+^CD25^+^FoxP3^+^ Tregs, the increase of hepatic CD8^+^CD25^+^FoxP3^+^ Tregs and CD3^+^ NKT cells and further reversed the HFD‐induced hepatitis [88]. Mammalian target of rapamycin complex1 (mTORC1) is well‐known target for its regulatory effects on SREBP‐1c, the pivotal element involved in lipid biosynthesis, and regulates Treg cell differentiation [89, 90]. Then researchers deeply explored the possible effects of HFD on Tregs in the MAFLD mouse model and found that the suppression of Foxp3 caused the downregulation of CD4^+^Foxp3^+^ Treg cells and CD8^+^CD122^+^ T cells and excessive lipid biosynthesis through upregulating the SREBP‐1c pathways‐related protein. Notably, phloridzin, extracted from apple trees and other plants, restored the function of Treg cells and ameliorated abnormal lipid metabolism by inhibiting the mTORC1/SREBP‐1c pathway in HFD‐diet mice and aP2‐SREBF1c transgenic mice [91].

With the development of research technology, more and more knockout animals (gene deletion) have been used to verify the relationship between specific genes and the pathogenesis of MAFLD. Costimulatory molecules B7.1 and B7.2 are mainly expressed on APCs and activate Tregs via interacting with CD28 on T cells [92]. Due to the HFD irritation, B7.1/B7.2 double‐deficient mice manifested liver inflammation, steatosis, and metabolic dysregulation due to the decreased number of Treg cells and the increase of M1‐type macrophages. Interestingly, the ACT of Treg cells did not improve MAFLD in a B7‐Lacking environment [92]. TLR7, as an endogenous receptor, can activate IFNs and proinflammatory cytokines (TNF‐α) signaling thus inhibiting the activity of Treg cells [93]. Compared with WT mice, TLR7 or IFN‐α/β receptor 1 knockout mice were more tolerant to hepatocyte death and steatohepatitis, and TLR7 antagonist also reversed the apoptosis of Treg cells afterward improved MAFLD symptoms [93]. Notably, recombination activating1 (Rag1)‐deficient (*Rag1* ^*−/−*^) mouse is regarded as a model of the ablative adaptive immune response including T/B cells [94]. The *Rag1* ^*−/−*^ mice fed with HFHC‐diet displayed more severe inflammation and immune microenvironment disturbance as evidenced by the significant upregulation of M1 macrophages and NK cells as well as the downregulation of anti‐inflammatory M2 macrophages. Janine Dywicki et al. further indicated that ACT of Tregs therapy increased Treg cells quantity but simultaneously exacerbated inflammation and even led to colitis in *Rag1* ^*−/−*^ mice fed with HFHC diet [95]. Similarly, another study has also indicated that although ACT of Tregs increased Treg cells in subcutaneous adipose tissues and decreased Th1 cells in livers, it caused more immune destruction and intensified hepatic steatosis in the HFHFD‐induced mice [96]. These findings reveal that the numbers and functions of Tregs in individuals may determine whether they override any beneficial effects of MAFLD therapy.

Except for the above factors, aging is also one of the pivotal factors affecting MAFLD development. It has been reported that aging mice exhibited the remarkable increase of splenic Treg cells, Type I regulatory (Tr1)‐like cells and systemic IL‐10 levels as well as decreased expression of inflammatory mediators (CCL2, CCL4, IL‐6, and TNF) to temper the severity of obesity‐associated metabolic derangements after HFD feeding [97]. As is known to all, aging is also intimately involved in cell carcinogenesis. However, it is worth noting that Treg disorder is attributed to the development of MAFLD‐associated HCC (MAFLD‐HCC) from MAFLD. The clinical data showed that patients with moderate MAFLD‐HCC had a higher level of Treg cells to suppress host antitumor immunity and dramatically facilitate the initiation and progression of cancer [98]. Butyrate, a metabolite of gut microbiota from MAFLD‐HCC patients, led to Treg cells amplification meanwhile reduced CD8^+^ T cells and B cells quantity via enhancing Foxp3 expression and restraining proinflammatory cytokines to reinforce immunosuppressive response of peripheral blood mononuclear cells (PBMC) from non‐MAFLD individuals [98]. On the other hand, during the process of MAFLD to HCC, neutrophil extracellular traps (NETs)‐related metabolic reprogramming impacted CD4^+^ T cells differentiation via the upregulation of Treg differentiation and function‐related genes. Surprisingly, depleting Tregs dramatically inhibits HCC initiation and progression in MAFLD models [99]. Again, Treg cells seem to play a bidirectional and complex role in the MAFLD progression than what we think.

### Effects of Th17–Treg cells interaction on MAFLD

Th17 cells and Treg cells are from the same precursor cells: the formers are generally considered to promote autoimmunity and tissue damage, while the latter antagonize excessive immune response and liver injury. Victoria Cairoli et al. clarified that the increased ratio of Th17 cells and Treg cells aggravated inflammation and even promoted liver fibrosis in the moderate and severe MAFLD adults [100]. Interestingly, after bariatric surgery, the ratio of Th17/rTreg cells in MAFLD patients was significantly altered, as evidenced by the decreased production of Th17 cells and IL‐17 as well as the increased production of Treg cells in the peripheral blood [101]. Since this clinical data directly points out that the imbalance of Th17/Treg cells is closely relevant to the development of MAFLD diseases [102], a variety of MAFLD animal models have been constructed to thoroughly investigate the effects of Th17/Treg cell imbalance on the pathogenesis and therapeutic drug development of MAFLD. The sodium nitrite‐induced chronic intermittent hypoxia (CIH) triggered the accumulation of hypoxia‐inducible factor 1 alpha (HIF1α) and activation of mTOR signaling, causing the decreased ratio of Treg/Th17 cells and exacerbating MAFLD in HFD‐diet mice [103]. Consistent with the previous studies about Th17 cells, microRNAs are also involved in the homeostasis of Treg cells through targeting their downstream genes. CD40, as a surface antigen of T cells, is the downstream regulatory elements of miR‐195. In HFD‐induced MAFLD rats, the remedy of miR‐195 overexpression and silencing of CD40, respectively, upregulated the expression of Treg cells and downregulated the proliferation of Th17 cells to maintain Th17/Treg cell balance as well as restrained the expression of Pro‐inflammatory factors, which subsequently improved the development of inflammation and MAFLD [104]. The FOS gene, as the target of microRNA‐29c and a gene suppressor, is tightly associated with the regulation of IL‐17 signaling pathway [105]. MCD‐induced MAFLD mice exhibited an increase of Th17 cells and the decrease of protective Treg cells through the high expression of FOS. In contrast, overexpression of microRNA‐29c could reverse the ratio of TH17/Treg cells and improve hepatic fat deposition by suppressing FOS/IL‐17 signaling pathway [105]. Not only limiting to microRNA, polypeptides, and associated‐receptors of inflammatory factors are also associated with the progression of MAFLD. The carnosine and α‐lipoic acid (ALA) exerted the properties of reducing oxidative damage and improving lipid metabolism and protected hepatocytes by inhibiting cell apoptosis in high‐calorie choline deficiency diet (HCCDD) of male rat models, which were attributed to the elevation of Treg/Th17 cells and IL‐10/IL‐17A ratio [106]. Li et al. further explained CXCR3 function on T cells and found that the administration of monokine induced by IFN‐γ (MIG)/CXCL9 disrupted Th17/Tregs balance by promoting CXCR3 expression on Th17 cells in a dose‐dependent manner and further exacerbated MAFLD in MCD‐diet mice, which was reversed by silencing of MIG/CXCL9 [107].

**Figure 3:**
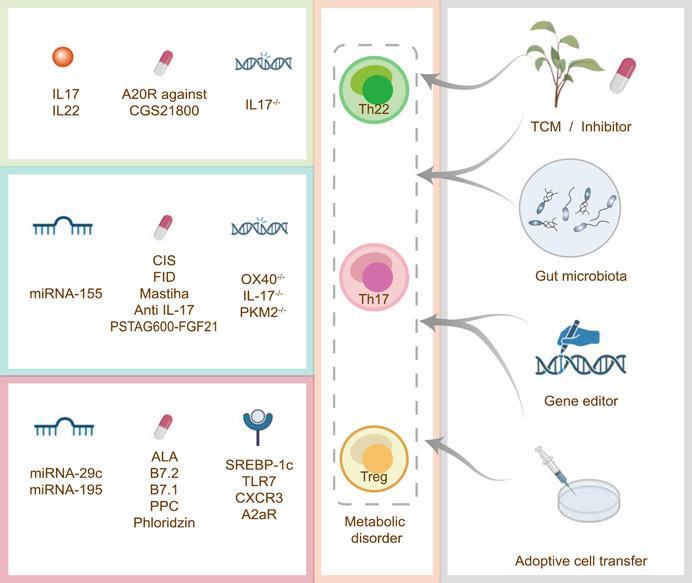
Changing the balance of Th22‐Th17‐Treg cells is one of the crucial approaches to deteriorate and improve MAFLD. The imbalance of Th22‐Th17‐Treg cells homeostasis exacerbated inflammation and fibrosis, or even gut dysbacteriosis by proinflammatory related factors, but was reversed through the treatment of drugs and microRNA, especially traditional Chinese medicine, probiotics, gene editor, or adoptive cell transfer (ACT)

After identifying the different regulatory mechanisms, many studies have begun to find specific drugs that targeted the above‐related links. Interestingly, the therapy of A2aR agonist improved the abnormal ratio of Th17/Treg cells as well as lipotoxicity by inhibiting the negative PTEN in palmitic acid (PA)‐cultured hepatocytes [66]. Similarly, the treatment with polyene phosphatidylcholine capsules (PPC) adjusted the imbalance of Th17/Treg cells, increased the mRNA level of FoxP3, and decreased Th17 cell‐related cytokines (IL‐6, IL‐17, and IL‐23) and the hepatic mRNA levels of RORγt and STAT3 to ameliorate liver inflammation and fibrosis in HFD‐induced MAFLD mouse model [108]. Growing evidence demonstrated that traditional Chinese medicine and its extracts possess the multicomponent and multitarget characteristics that can improve immune cell imbalance and are widely used for the treatment of MAFLD in clinical and basic research. Research further showed that Xiaochaihu decoction, recorded from the *Shang‐Han‐Lun*, effectively mediated IL‐17 function and improved MAFLD by influencing multiple signaling pathways including AKT1, IL‐6, JUN, MAPK8 and STAT3 [109]. In addition, Yun Liu et al. confirmed that 3, 3'‐diindolylmethane (DIM), the abundant ingredient in cruciferous vegetables, shifted the Treg/Th17 cells imbalance and ameliorated intra‐hepatic lymphocyte infiltration via the activation of aryl hydrocarbon receptor (AhR) signaling, which was attenuated by AhR antagonist CH223191 while was enhanced by anti‐TLR4 neutralizing antibody [110]. Another study reported that the HFD remarkably increased the expression of Pro‐inflammatory adipokine such as chemerin, chemerin‐like receptor 1 (CMKLR1) and Pro‐inflammatory C‐C chemokine receptor 2 (CCR2) to regulate the intrahepatic ratio of Treg/Th17 cells in rats. Meanwhile, berberine (BBR), the main component of the Chinese medicine *Coptis chinensis*, obviously improved free fatty acid (FFA) accumulation, lipid deposition, and steatohepatitis in rats by repairing the balance of Treg/Th17 ratio and downregulating the chemerin/CMKLR1 pathway [111]. Similarly, Koumine, an indole alkaloid, restrained inflammatory cell infiltration and ameliorated the adipose‐like lesions of hepatocytes in HFD‐induced MAFLD rats by decreasing the secretion of Th17 cells and their cytokines and increasing the levels of Treg cells and anti‐inflammatory cytokine IL‐10 [57]. Likewise, Chinese herbal formulas are also very effective means of preventing MAFLD in the clinic. It has been reported that Dahuang Zhechong pills (DHZCPs) therapy, as a TCM ancient formula, maintained the balance of Th17/Treg cells and regulated the levels of inflammatory cytokines, IL‐17, IL‐10, and TNF‐α to improve liver function in MAFLD patients [112]. Intestinal microbiome disorder is also the critical element of Th17 and Treg cells‐related metabolic syndrome [113]. The gut microbiome as a MAFLD modulator opens a new direction for the treatment of MAFLD. CB, the probiotics in the gut, inhibited intestinal hepatitis immune disorder and protected the liver away from damage by significantly promoting the differentiation of CD4^+^ T cells into Tregs and increasing the levels of Foxp3, IL‐4 and IL‐22 as well as reducing the differentiation of CD4^+^ T cells into Th17 and the expression of IFN‐γ and IL‐17 in liver and ileum of HFD‐induced mouse model [63]. Hence, repairing the imbalance of Th17/Treg cells rather than simply increasing the number of these immune T cells is a potential therapeutic direction for MAFLD (Figure 3).

### Effects of CD8^+^ T cells on MAFLD

**Figure 4:**
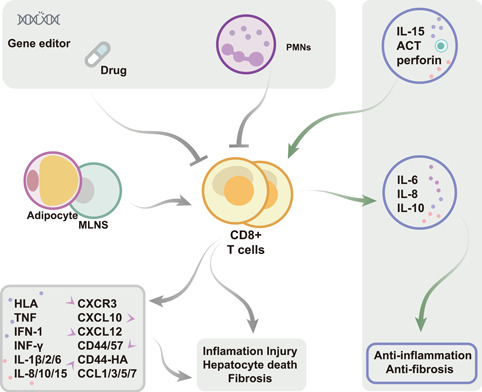
CD8^+^ T cells play different regulatory roles and influence metabolic dysregulation. CD8^+^ T cells migrated to the liver and promoted diet‐induced MAFLD through binding to the Pro‐inflammatory chemokine receptor axis or affecting other immune cells (macrophages, neutrophils) response, but inflammatory damage was ameliorated by treatment with several anti‐inflammatory drugs. However, in special cases, CD8 + T cells inhibited fibrosis and even improved MAFLD either by restoring the balance of gut microbiota or combination therapy with drugs

Based on the cluster of differentiation T‐lymphocytes, CD8^+^ T cells were recently found to infiltrate in the liver of MAFLD patients together with a higher degree of steatosis, similar to the Th cells that were derived from CD4^+^ T cells [114]. Another clinical study showed the higher frequencies of IL‐7Rα^low^ CD8^+^ T cells and IL‐7Rα^low^CX3CR1^+^CD8^+^ T cells but not CD4^+^ T cells in the serum of obese children, compared to the children without metabolic syndrome [115]. Besides, Magar Ghazarian et al. further reported that these accumulated and activated pathogenic CD8^+^ T cell subsets were largely supported by IFN‐I responses in the liver, which promoted insulin resistance and caused metabolic disturbances in HFD‐induced mouse model. Either IFNαR1 inhibitors or IFNαR1 deficiency improved metabolic parameters in these mice [116]. Except for the HFD model, increased CD4^+^ T cells and CD8^+^ T cells were also detected in the iron‐supplemented chow diet‐induced MAFLD mice [117]. Furthermore, antigen peptide transporter 1 (TAP1) can transport antigen to I MHC and conduce to the generation of CD8^+^ T cells. HFD‐diet *TAP1* ^*−/−*^ mice (without CD8^+^ T cells) resulted in the improvement of hepatic steatosis even though it was accompanied by increased hepatic CD4^+^ T cells [118]. Collectively, several of the above cases revealed that the morbidity of MAFLD may be tightly associated with the activation of CD8^+^ T cells (Figure 4). As a result, many investigators have embarked on the exploration of drugs by inhibiting CD8^+^ T cell activation for MAFLD therapy. Lunasin (an anti‐inflammatory peptide) and cardioaid (a phytosterol) both effectively reduced serum TG levels and modulated immune homeostasis via the suppression of the CD4^+^/CD8^+^ cells ratio in the HFD‐treated mice [119]. Additionally, astaxanthin, an antioxidant carotenoid, was found to effectively ameliorate MAFLD and fibrosis by reducing intracellular TG content in hepatocytes, inhibiting the expression of liver fibrosis‐related genes, suppressing hepatic CD4^+^ and CD8^+^ T cell accumulation as well as Kupffer cell infiltration in HFD‐induced MAFLD mice [120]. Programmed cell death 1 (PD‐1) and its ligand PD‐L1 expressed on all T cells are critical checkpoints of auto‐aggressive tissue damage. It was found that the increased axis of PD1/PD‐L1 inhibited CD8^+^ T cell activity and alleviated total CD8^+^ T cell‐mediated immune damage in patients with steatohepatitis [121]. Wabitsch et al. also revealed that the combination with metformin improved the therapeutic effect of anti‐PD‐1 in steatohepatitis of HCC by impairing motility and metabolic function of CD8^+^ T cells [122].

In addition to pharmacological interventions, more researchers wonder whether the key receptors expressed on CD8^+^ T cells could play a positive role in MAFLD by restraining their function. In patients with simple steatosis, the decrease of TLR9 expression could prevent MAFLD and liver damage by increasing a low level of CD8^+^ T cell, IFN‐γ secretion, and kupffer cells activation. In contrast, patients with steatohepatitis could not automatically initiate a TLR9‐related protection adaptation to modulate hepatocellular injury [123]. Remarkably, the chemokine receptor CCR2 and leukocyte adhesion molecule CD44 were all increased in MAFLD patients and contributed to lipid‐mediated hepatitis progression. After being fed with lithogenic diet, CCR2^−/−^ mice were totally protected from hepatitis, but CD44^−/−^ mice were only partly recovered. In the absence of CCR2, CD44‐mediated hyaluronic acid (HA) binding was not increased on CD8^+^ T cells during lithogenic diet feeding [124]. Other researchers have also noted the immune crosstalk between endothelial cells and CD8^+^ T cells through CXCL12–CXCR4 axis. Either CXCR4 antagonist or CXCL12‐neutralizing antibody could reverse the high expression of CXCL10, CXCL12 and human leukocyte antigen (HLA) I, which reduced the transendothelial migration of CD8^+^ T cells to attenuate endothelial injury in MAFLD patients [125]. Unexpectedly, it is reported that CD8^+^ T cells seem to have anti‐inflammation or anti‐fibrotic effects in ways, not just aggravating inflammation. It is speculated that the immune deficiency of CD4^+^ cells and CD8^+^ T cells contribute to trigger MAFLD. Oral administration of an extract containing ergosterol (a precursor of vitamin D2) increased the level of hepatic CD4^+^ T cells and CD8^+^ T cells, decreased the level of Pro‐inflammatory cytokines and circulating lipid content, and improved hepatocytes ballooning caused by HFD [126]. Recently, Breuer et al. displayed that CD8^+^ T cells inhibited liver inflammation and HSC activation through the secretion of IL‐10 in both patients with moderate MAFLD and in vitro models of obesity and hyperlipidemia‐induced steatohepatitis [127]. Additional research also showed that tissue‐resident memory CD8^+^ T cells (Trm) were activated by IL‐15 to improve steatohepatitis, attract HSCs in a CCR5^−^dependent manner and induce apoptosis of HSCs via inducing FasL‐Fas pathway in HFD mouse model [128].

In addition, CD8^+^ T cells also modulate the progression of MAFLD by affecting other immune cells rather than function alone in many cases. For instance, circulating neutrophils or polymorphonuclear neutrophils (PMNs) are positively correlated with hepatic steatosis and hepatocellular ballooning in clinical cases. Interestingly, the activation and proliferation of CD4^+^ cells and CD8^+^ T cells were significantly inhibited in cocultured PMNs of moderate and severe steatohepatitis patients and PBMCs [129]. The diet‐induced obesity and the administration of bromodichloromethane (BDCM) caused metabolic oxidative stress, accelerated the steatohepatitis progression in mice by increasing the CD57 expression in CD3^+^CD8^+^ T cells along the CYP2E1‐leptin axis, further mediating the release of proinflammatory cytokines, such as IL‐1β, IFN‐γ, and IL‐2 in the liver [130]. Moreover, as a crucial element in the immune system, adipocytes‐derived leptin promoted the release of IFN‐γ and granzyme B from hepatic CD8^+^ T cells in HFD‐induced MAFLD mice, thus promoting pyroptotic‐like cell death of hepatocytes or macrophages [131]. In particular, it has been found that macrophages and CD8^+^ T cells also synergeticly influenced the immune microenvironment of MAFLD. In early steatohepatitis, the infiltration of macrophages occurred predominantly and was accompanied with the increase of TNF and IL‐1β expression, whereas in progressed steatohepatitis, the inflammatory infiltration of CD8^+^ T cells prominently intensified and accompanied by increasing IL‐6 and IL‐8 level in portal vein of patients [132]. Ping Lu et al. further confirmed this phenomenon and innovatively found that the increased infiltration of macrophages and CD8^+^ T cells as well as phenotypic transformation from M2 to M1 macrophages were also observed with Lep^ΔI14/ΔI14^ model of spontaneous rat‐specific steatohepatitis [133]. Interestingly, transfer of perforin can regulate the activation and survival of CD8^+^ T cells, inhibit the hepatic infiltration of Pro‐inflammatory M1 macrophages and CD8^+^ T cells‐mediated proinflammatory phenotype transformation, resulting in the improvement of MCD‐ and HFD‐induced steatohepatitis [134]. Similarly, lycopene effectively improved hepatic oxidative stress, reduced liver fibrosis, and liver inflammation by inhibiting NADPH oxidase expression, CD3^+^ and CD8^+^ T cell accumulation, as well as decreasing M1 macrophage levels [135]. On the other hand, mice with Ath‐diet‐induced metabolic disorders were intravenously administered with M2 macrophages treated by baicalin, which reduced the quantity of CD4^+^CD25^−^ T and CD8^+^CD25^−^ T cells in peripheral blood, decreased hepatic T cell infiltration, and alleviated the liver injury [136].

It is remarkable that impaired intestinal immune function and increased intestinal permeability may interfere with the cross‐talk between the gut and the liver, further aggravating MAFLD due to changes of gut microbiota. Clinical studies have shown that MAFLD patients were observed with a decrease of CD4^+^ and CD8^+^ T lymphocyte and dysbiosis of the intestinal flora [137]. In a study of MAFLD‐related intestinal immunity, CCL5‐induced migration of MLN cells promoted the activation of CD4^+^ T cells and CD8^+^ T cells and reduced the proportion of memory CD4^+^ T cells and CD8^+^ T cells in liver, aggravating inflammatory liver injury [138]. The activation of enteric CD8^+^ T cells could preferentially home in to the liver and then affect the local immune environment [139]. Clinical trials have also shown that probiotics sachet (MCP® BCMC® strains) protected liver against the reduction of intestinal villous CD8^+^ T cells and significantly improved hepatic steatosis in patients with mild, moderate, and severe MAFLD [140].

### Effects of γδT cells on MAFLD

The γδT cells, double negative T cells, account for only a small proportion of total T lymphocytes, but have different proinflammatory and anti‐inflammatory functions in the immune system. More importantly, it has been found that, compared to peripheral blood, the ratio of γδT cells is abundant in the liver and plays a crucial role in liver immunity. For instance, the IFN‐γ‐producing γδT cell subset (γδT1) cells eliminated the activated HSCs by enhancing cytotoxicity [141]. Additionally, the latest research indicated that the subset of TCR γδT cells aggravated IL‐17A secretion to facilitate liver inflammation by NF‐κB signaling pathway in MAFLD under arachidonic acid stimulation [142]. The γδT cells were recruited to the liver by CCR2/5 and accelerated the expansion and differentiation of CD4^+^ cells by upregulating CD1d, which resulted in the hepatic steatosis and leukocytic infiltration in MCD‐induced mouse model [143]. On the other hand, in HFD‐induced mice, hepatic γδT‐17 cells secreted high levels of IL‐17A and reacted with lipid antigens of microorganisms presented by CD1d to exacerbate MAFLD, which was ameliorated by Abx treatment or CD1d knock out [144]. Zhou et al. further found that FMT from mice fed with a standard chow diet could effectively decrease the IL‐17 levels and regulate intrahepatic immune in HFD‐fed mice by upregulating Foxp3, IL‐4, and IL‐22 [145]. Recent studies have shown that herbal medicine has significant therapeutic effects on MAFLD based on the gut‐liver axis. A traditional Chinese formula, Yijin‐Tang (YJT) effectively improved the composition of hepatic lipid metabolites and gut microbiome and improved MAFLD injury in HFHC‐diet mice, which was highly associated with the reduction of γδT cells [146]. Collectively, these results suggest that γδT cells may become the potential targets of MAFLD but need further exploration.

### Effects of NKT cells on MAFLD

**Figure 5:**
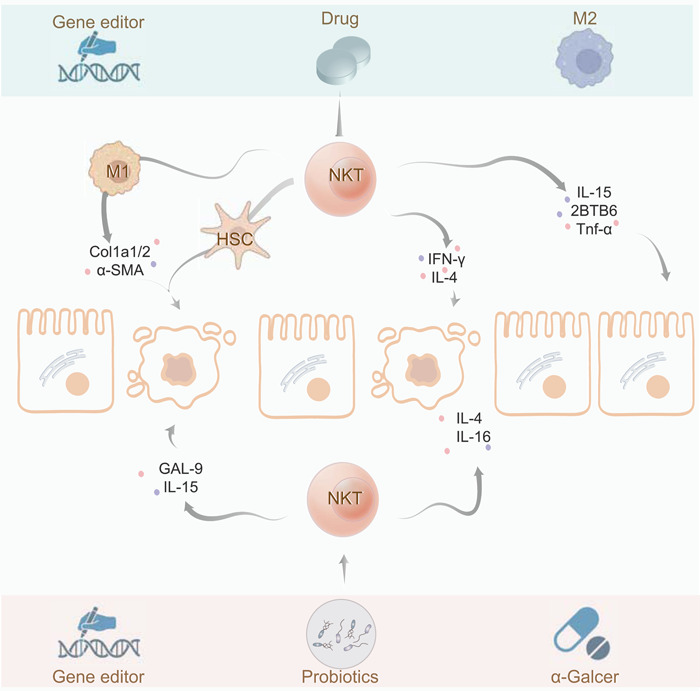
NKT cells exert both positive and negative effects on MAFLD. NKT cells have more complex immune properties and are a double‐edged sword for MAFLD. Even in the same gene knockout animal model of MAFLD, NKT cells also show contrasting experimental results that are the promotion of lipid accumulation and inflammatory infiltration by proinflammatory immune cells and factors, but on the other hand, the inhibition of MAFLD by anti‐inflammatory factors. Gene editing, drugs and probiotics are effective ways to improve metabolic damage via regulating NKT cells. Some materials of the illustrations are quoted from BioRender.

NKT cells are present in epithelia and lymphoid organs and are involved in the immune regulation of metabolic disorders by regulating HSC activation and inflammatory cells infiltration [32]. Previous studies pointed out that hepatic NKT cells secreted hedgehog (Hh) ligand that directly induced osteopontin (OPN) expression in a paracrine manner and promoted myofibroblastic activation [147]. Later, a double‐blind clinical trial with oral administration of β‐glucosylceramide (GC) ameliorated steatohepatitis by decreasing insulin resistance level, hepatic TG level and content of CD4^+^ T cells and NKT cells in the moderate MAFLD patients [148]. These studies revealed that the regulation of NKT cells might play a decisive role in the MAFLD progression (Figure 5). In addition, a variety of studies generally reveal that the function of NKT cells in MAFLD may be associated with different cytokines and chemokine receptors. The proinflammatory cytokine LIGHT (TNFSF14) aggravated hepatic inflammation in the HFHC diet by increasing the Zinc finger and BTB domain containing 16 (ZBTB16), which was the critical transcription factor for maintaining NKT cell function [149]. Proinflammatory chemokine receptor (CXCR16) also promoted hepatic NKT cell accumulation, thereby controlling macrophage infiltration and the increase of inflammation factors (TNF and MCP‐1), leading the worsen of hepatic steatosis [150]. Besides, it was found that CXCR6 promoted iNKT migration in the liver and the release of proinflammatory factors IFN‐γ and IL‐4 from iNKT cells, which further resulted in macrophage infiltration and hepatic inflammation in the livers of MCD diet‐treated mice [151].

Usually, gene‐knockout animal models have gradually become one of the common methods to study the function of different immune cells, and NKT cells are no exception. Interestingly, the deficiency of CXCR6 improved MAFLD and protected fatty liver far from ischemia–reperfusion injury (IRI) by reducing the recruitment of hepatic NKT cells and production of inflammatory cytokine IFN‐γ in the HFD‐treated mice [152]. Similarly, the deficiency of PTP1B also significantly ameliorated the MCD‐induced MAFLD accompanied a decrease in number of NKT cells and increased the number of M2‐type macrophages [153]. On the other hand, knockout of typical inflammatory factor like NF‐κB1 also triggered the recruitment of NKT cells by early overproduction of IL‐15 as well as increased the level of IFN‐γ and OPN in MCD‐fed mice [154]. Unexpectedly, although IL‐15 was important for the production and maintenance of NKT cells, the specific deletion of IL‐15 in hepatocytes and macrophages did not significantly affect hepatic lipid accumulation [155]. Therefore, there may exist many factors that affect the proliferation and differentiation of NKT cells, not only IL‐15. An invariant alpha‐chain Jalpha 18 is the main component of TCRs of iNKT thus Jα18‐knockout mice have been used as iNKT cell‐deficient animal models [156]. After feeding with choline‐deficient l‐amino acid‐defined (CDAA) diet, decreased expressions of hepatic inflammatory factors, such as TNF‐α, IFN‐γ, IL‐6, IL‐17A, and CXCR3, and inflammasome activation‐related genes such as NLRP3 were obtained in Jα18 knockout (Jα18^−/−^) mice but not in the WT mice [157]. As is well‐known, the development of NKT cells depends on the antigen presentation molecule CD1d expressed on thymocytes. An HFHC diet promoted the degree of NKT cells infiltration in the liver, while NKT deficiency (Cd1d^−/−^ mice) reduced hepatic lipid content, the hepatic expression of fibrosis‐related genes like Col1a1, Col1a2 and α‐SMA, as well as CD11b‐positive macrophage infiltration [158]. Similarly, the lipid metabolic disorders‐caused MAFLD were associated with the increase of hepatic NKT cells but were improved in CD1d^−/−^ mice [159], suggesting a critical role of NKT deficiency in preventing MAFLD progression.

Sometimes, even though the researchers used the same knockout mouse models, they still get contradictory results about the effects of NKT cells on MAFLD. Recent study has demonstrated severe fat accumulation, metabolic alterations, and the increase of inflammatory mediators, such as IL‐6, MCP‐1 in livers of HFD‐fed CD1d^−/−^ mice [160]. It coincides with the above that KK‐A(y) mice, as a model of metabolic deficiency, have a reduced fraction of hepatic NKT cells after feeding with HFD diet [161]. Notably, it has been reported that Jα18^−/−^ led to lipid metabolism disorders, steatohepatitis, and liver fibrosis via the inhibition of NKT cell differentiation in MCD‐fed mice, which was effectively reversed by ACT of iNKT cells into Jα18^−/−^ mice [162]. In addition to gene editing, intraperitoneal injection of α‐GalCer (NKT activator) was reported to decrease the levels of hepatic proinflammatory CD4/CD8^+^ T cells and cytokines (IL‐2, IL‐6, IL‐17A, TNF‐α, and IFN‐γ) while increased the levels of iNKT cells and anti‐inflammatory cytokines (IL‐4 and IL‐10), especially iNKT2 cells, maintaining hepatic homeostasis and improving hepatic steatosis in HFD‐induced MAFLD [61]. The interaction of T cell Ig and mucin domain (Tim)−3 with its ligand, galectin‐9 (GAL‐9) can regulate immune cells like NKT cells and maintain hepatic cell homeostasis. Treatment with galectin‐9 could not only Tim‐3(+) NKT cell apoptosis and induce the depletion of NKT cells, but also promote NKT cell proliferation and improve hepatic steatosis by interacting with Tim‐3‐expressing Kupffer cells and subsequent secretion of IL‐15 in HFD‐fed model [163]. Interestingly, the specific deletion of macrophage migration inhibitory factor (MIF) improved metabolic disorder and liver fibrosis by reducing the expression of pro‐fibrosis genes and inducing the polarization and activation of NKT cells [164]. Besides, another article pointed out that NK and iNKT cells directly killed HSCs and protected against hepatic steatosis and fibrosis by NKG2D signaling [165]. It was also found that HFHC diet‐associated orthotopic and spontaneous MAFLD‐HCC was associated with NKT cell dysfunction caused by elevated cholesterol signaling. Rosuvastatin, an anticholesteremic agent, could restore NKT amplification and cytotoxicity by suppressing mTORC1/SREBP2‐driven excessive hepatic cholesterol synthesis [166]. Notably, bacterial flora also plays an important role in the treatment of MAFLD. A combination of probiotic bacteria (*bifidobacteria, lactobacilli, and Streptococcus thermophilus*) was found to improve hepatic lipid accumulation as well as insulin resistance in mice by increasing NKT cells [167]. The lipid antigens on the probiotics surface facilitated proliferation of hepatic NKT cells, indicating that the regulation of gut microbiota is a potential treatment of MAFLD.

### Effects of MAIT on MAFLD

MAIT cells specifically recognize the antigens of microorganisms and are account for only about 5% of blood T cells but are up to 50% of all T cells in the human liver. Hence, investigators turned their attention to the relationship between MAIT cells and MAFLD [168]. The nonclassical I MHC molecule MR1 can capture and present antigens to MAIT cells and trigger their activation [169]. It was noted that MAFLD patients also had a decreased level of MAIT cells in the peripheral blood but an increase of MR1 expression in Kupffer cells. Under the induction of MCD, MAIT cells were recruited to the liver and activated in an MR1‐dependent manner to induce polarization of M2 macrophages and improve hepatic steatosis in MAFLD mice [170]. Conflictingly, Toubal et al. found that MAIT cells impaired lipid metabolism in adipose tissue by inducing M1 macrophage polarization in an MR1‐dependent manner, and led to intestinal metabolic dysfunction by inducing intestinal flora dysregulation and increasing permeability [171]. Additional evidence also suggested that the MAIT cells exhibited an inflammatory profile, changed immune cell homeostasis and promoted inflammation in visceral adipose tissues and ileums of HFD‐fed mice [172]. Emerging evidence demonstrated that the human MAIT cells isolated from PBMC were significantly decreased in MAFLD patients with the identification of CD3^+^CD4–CD161^hi^Vα7.2^+^, compared to healthy groups. Furthermore, coculture with the human MAIT cells and human hepatic myofibroblasts (HMFs) experiment found that the secretion of TNF from activated MAIT cells could increase the production of IL‐6 and IL‐8 in HMFs, enhance the activity of myofibroblasts and aggravate MAFLD progression [173]. This study reveals that different cellular triggers could induce the opposite immune effects of MAIT cells on MAFLD, providing a new direction for disease therapy.

## DISCUSSION

The main clinical manifestation and pathogenic mechanism of MAFLD is dyslipidemia and the abnormal increased fat in the liver. Therefore, previous and most current research focused on the regulatory role of glycolipid metabolism but not T cell immune dysregulation in MAFLD development. As time went by, researchers gradually observed the accumulation of inflammatory immune cells like Th1 cells, M1 macrophages, Th17 cells, CD8^+^ T cells and γδT cells in the liver or adipose tissues while the decrease of the quantity and dynamism of anti‐inflammatory cells such as Th2 cells, Treg cells and NKT cells both in clinical and animal studies. These observations imply that the destruction of immune homeostasis may cause hepatic inflammatory responses, liver fibrosis or further hepatocarcinogenesis through mediating inflammatory cytokines‐ and chemokines‐related signaling pathways.

Interestingly, a variety of studies have revealed that, due to the complex regulatory properties of cells, same immune cells may perform opposite functions in different or even same animal models of MAFLD. For instance, the infiltration of Th2 cells in Ath‐fed TLR9‐deficient mice induced the resistance of BM macrophages to M1 activation and then inhibited lobular inflammation and MAFLD progression [59], while caused severe MAFLD and liver fibrosis in HFD‐induced obese Alms1 mutant mice [51]. The reason for this discrepancy is presumed due to the inability of current models to fully mimic the representative human MAFLD. Th22 cell infiltration also exacerbated hepatocyte lipotoxicity in response to MCD stimulation but ameliorated liver inflammation in the IL‐17‐deficient mouse model [67]. It may be directly related to an antagonistic relationship between Th1 cells and Th2 cells or Treg cells and Th17 cells while a synergistic relationship between Th1 cells and CD8^+^ T cells or Th1 cells and Th17 cells. Meanwhile, several subsets of T cells rather than the single cell secreted multifarious cytokines, which in turn act on themselves and further lead to a more complex immune response. Interestingly, unlike the effects induced by Th cells, activated hepatic CD8^+^ T cells commonly cause local inflammation but are also able to inhibit liver fibrosis and steatosis in particular situations [128]. Presumably, it is possible that certain cytokines specifically activate one subset of CD8^+^ T cells to exert their hepatoprotective effects. As the bridges between innate and acquired immunity, NKT cells act as a double‐edged sword that can either exacerbate steatohepatitis or improve MAFLD in different in vivo animal models. Likewise, MAIT cells are also demonstrated to improve lipotoxicity and hepatitis, but also result in local tissue inflammation and even liver fibrosis. It is assumed that NKT cells or MAIT cells are stimulated by specific factors and then differentiate into diverse functional subsets during the process of MAFLD. Of course, different consequences caused by the multiple complexity of immune cells cannot be excluded. It is particularly important to note that individual immune cells have also been found in previous studies to act contradictoryly in different liver diseases. For example, Treg cells play a protective role in MAFLD, but enhance immunosuppression to exacerbate HCC in the MAFLD‐HCC process, possibly due to the reprogramming effects of Treg cells [99].

Combined with these results, we need to focus on the regulatory role of the entire immune microenvironment rather than individual cells or inflammatory factors played in the progression of MAFLD. Furthermore, immune crosstalk causes an array of changes in the hepatoenteral circulation and adipose tissue, leading to more severe hepatic inflammation [174]. Clinical cases have shown that diet‐induced fat accumulation disrupted intrahepatic T‐cell homeostasis (including Th17 cells, Treg cells, NKT cells, and CD8^+^ T cells) and further aggravated MAFLD [100]. Another study has also found that high calories were associated with abnormal T cell glycolysis and proliferation [175]. In addition, it has been reported that MAIT cells broke intestinal integrity by inhibiting the function of *bifidobacteriaceae* (*Actinobacteria* phylum) and *lactobacillaceae* (*Firmicutes* phylum) families and aggravated inflammation by inducing polarization of M1 macrophages in the obese mice [171]. Furthermore, both dietary intervention and aerobic exercise intervention participants showed improved MAFLD parameters, which were associated with a significant reduction in intrahepatic MAIT cells numbers [176]. These results suggest that genetic and environmental factors as well as immune cell metabolism may be the main factors affecting T cell function in MAFLD progression. Moreover, abnormal T cells tend to result in or aggravate several immune‐related diseases that have a higher incidence of MAFLD. In addition to the immune T cells mentioned above, follicular helper CD4^+^ T cells (TFH cells) and the molecules secreted by TFH cells provide help to B cells in humoral immunity [177]. However, pTFH cells response in MAFLD patients with BMI > 35 and BMI < 35 at baseline and postvaccination was no difference [178]. Therefore, the function of TFH cells is still to be explored in MAFLD. Another concern remained that AIH has similar inflammatory responses without hallmark pathological markers to MAFLD, which can also be aggravated by some anti‐MAFLD drugs [179]. Therefore, the complexity of immune regulation needs to be elucidated for exploring new effective drugs and treatments for MAFLD.

Another problem for researchers is that MAFLD is hardly detectable in the early stages and lacks a first‐line treatment in clinic. The complex immune regulation of T cells played in the MAFLD process is gradually gaining more attention. In addition, we believe that treatment strategies for MAFLD should be considered to focus more on how to restore healthy immune patterns rather than only simply induce or suppress the function or numbers of some specific immune cell types. Furthermore, Chinese medicine was regarded as a potential therapeutic modulator of immunity to improve MAFLD due to its multitargeted and multicomponent properties. For instance, Xiaochaihu decoction and Dahuang Zhechong pills all reconciled the balance of Th17/Treg cells and restrained the levels of inflammatory cytokines to effectively attenuate lipid accumulation and MAFLD progression [109, 135]. Furthermore, it is also noteworthy that traditional Chinese medicine can also improve the balance of intestinal flora. For instance, inulin originating from plants alleviated diet‐induced barrier dysfunction by increasing antimicrobial peptides and decreasing the luminal bacteria of Ileum in western‐style diet (WSD) mice [180]. Tianhuang formula chiefly consists of *Panax notoginseng* and *Coptis chinensis*, and reshaped the intestinal flora such as increasing *Lactobacillus* and its metabolites to improve HFD‐induced MAFLD [181]. In addition to being influenced by TCM, the gut microbiota is closely related to the immune microenvironment and also has the potential to become a possible effective treatment approach for MAFLD [182, 183]. Microbiota‐targeted interventions (soluble dietary fiber and *Clostridium butyricum*) are the effective regimens to increase microflora diversity and improve chronic inflammation in MAFLD [184]. There is evidence that healthy intestinal bacteria are involved in alleviating gut disorders and regulating immunity, so FMT has the potential to become a hopeful and essential therapy for MAFLD in the near future.

## CONCLUSION

In summary, we first reviewed the pathogenesis of MAFLD and the function of different T cells. Second, we deeply summarized and analyzed how different T cells interact with each other to participate in the development of MAFLD. This current study not only points out the importance of immunomodulatory function of T cells in the process of MAFLD but also provides new ideas and breakthroughs for the discovery and development of further drug trials and mechanistic studies.

## AUTHOR CONTRIBUTIONS

Xiaojiaoyang Li conceived the original idea and supervised the study. Jia Liu, Mingning Ding, Jinzhao Bai, Ranyi Luo, Runping Liu, and Jiaorong Qu prepared the manuscript and figures. All data were generated in‐house and no paper mill was used. All authors have approved the final manuscript.

## CONFLICT OF INTEREST

The authors declare no conflict of interest.

## Data Availability

No new data and scripts were used in this paper. Supporting Information Materials (figures, tables, scripts, graphical abstracts, slides, videos, Chinese translated version, and updated materials) may be found in the online DOI or iMeta Science http://www.imeta.science/.
